# A novel risk prediction model for post-treatment ischemic stroke recurrence using plasma antithrombin III and thromboelastography

**DOI:** 10.5937/jomb0-61916

**Published:** 2026-04-15

**Authors:** Yi Cheng, Baobao Huang, Shan Jiang

**Affiliations:** 1 Huangshan Shoukang Hospital, Department of Transfusion Medicine, Huangshan, Anhui, China

**Keywords:** ischemic stroke, antithrombin III, thromboelastography, dual antiplatelet therapy, risk prediction model, ishemijski moždani udar, antitrombin III, tromboelastografija, dvostruka antitrombocitna terapija, model za predviđanje rizika

## Abstract

**Background:**

The primary aim of this investigation was to build and test a novel risk prediction model incorporating plasma antithrombin III (ATIII) activity and thrombelastography (TEG)-derived parameters. This approach seeks to enhance the ability to stratify the risk of stroke recurrence among ischemic stroke (IS) patients receiving dual antiplatelet therapy (DAPT).

**Methods:**

In this prospective cohort study, 200 consecutive patients diagnosed with non-cardiogenic IS were recruited during a one-year period (May 2024 to May 2025). All participants had their ATIII activity, TEG parameters, and coagulation function indicators tested within 24-72 hours of DAPT commencement. After initial variable screening via univariate analysis. A logistic regression-based risk model was built, with its ability to distinguish outcomes evaluated using the receiver operating characteristic (ROC) curve.

**Results:**

Recurrent events occurred in 21.00% (42/200) of cases within 3 months post-DAPT Multivariate analysis established ATIII, TEG-LY30, and D-Dimer as independent risk factors and TEG-MA as protective. The resultant model exhibited superior predictive power (A U C = 0.9480, 95% C I= 0.9l48~ 0.9813; sensitivity 90.48% , specificity 86.71%). Internal validation, yielding AUCs of 0.9521 in the training set and 0.9437 in the validation set, verified the model's strong generalizability. Subgroup evaluations further revealed the model's robust performance in both large artery atherosclerosis (AU C= 0.9505) and small vessel occlusion (AUC=0.9395) subtypes.

**Conclusions:**

Integrating ATIII activity with TEG parameters enables the development of a novel model to predict the risk of IS relapse following DAPT.

## Abstract (sr)

**Uvod:**

Primarni cilj ovog istraživanja bio je da se napravi i testira novi model za predviđanje rizika koji uključuje aktivnost plazma antitrombina III (ATIII) i parametre izvedene iz trombelastografije (TEG). Ovaj pristup ima za cilj da poboljša mogućnost stratifikovanja rizika od ponovnog moždanog udara kod pacijenata sa ishemijskim moždanim udarom (IS) koji primaju dvostruku antitrombocitnu terapiju (DAPT).

**Metode:**

U ovoj prospektivnoj kohortnoj studiji, 200 uzastopnih pacijenata sa dijagnozom nekardiogenog insuficijencije krvi (IS) regrutovano je tokom perioda od jedne godine (od maja 2024. do maja 2025. godine). Svim učesnicima je testirana ATIII aktivnost, TEG parametri i indikatori koagulacione funkcije u roku od 24-72 sata od početka DAPT terapije. Nakon početnog skrininga varijabli putem univarijantne analize, izgrađen je model rizika zasnovan na logističkoj regresiji, čija je sposobnost razlikovanja ishoda procenjena korišćenjem ROC krive (recirculator operating characteristic).

**Rezultati:**

Rekurentni događaji su se javili u 21,00% (42/200) slučajeva u roku od 3 meseca nakon DAPT-a. Multivarijantna analiza je utvrdila ATIII, TEG-LY30 i D-dimer kao nezavisne faktore rizika, a TEG-MA kao zaštitne. Dobijeni model je pokazao superiornu prediktivnu moć (AUC=0,948 0, 95%CI = 0,9148~ 0,9813; osetljivost 90,48% , specifičnost 86,71%). Interna validacija, koja je dala AUC od 0,9521 u skupu za obuku i 0,9437 u skupu za validaciju, potvrdila je snažnu generalizaciju modela. Evaluacije podgrupa su dodatno otkrile robusne performanse modela i kod podtipova ateroskleroze velikih arterija (AUC=0,9505) i okluzije malih krvnih sudova (AUC= 0,9395).

**Zaključak:**

Integracija ATIII aktivnosti sa TEG parametrima omogućava razvoj novog modela za predviđanje rizika od recidiva IS nakon DAPT-a.

## Introduction

Ischemic stroke (IS) stands as the world's second major cause of death and third main contributor to disability. China sees more than 3.4 million new IS cases diagnosed each year (based on the latest available data from Tu et al. [1]. This disease burden is expected to grow steadily as the population continues to age [1]. Secondary prevention is key to lowering the risk of IS recurrence, with dual antiplatelet therapy (DAPT) being recommended as the first-line option for patients with non-cardiogenic IS [2]. Nevertheless, a recurrence rate of 15-20% persists among DAPT-treated patients [3]. This highlights the urgent need for more precise risk stratification tools to identify high-risk populations and facilitate personalized treatment adjustments. The existing models for predicting post-DAPT recurrent risk in IS patients largely depend on a combination of clinical indicators (such as age, hypertension, diabetes, and National Institutes of Health Stroke Scale [NIHSS] scores), biomarkers (like high-sensitivity C-reactive protein [hs-CRP] and matrix metalloproteinase-9 [MMP-9]), or neuroimaging features (including infarct volume and severity of large vessel stenosis) [4][5]. Their predictive power, however, is constrained by several shortcomings. A key limitation is the dependence on static parameters (e.g., single-timepoint biomarker measurements or static imaging findings), which fail to reflect real-time fluctuations within the coagulation-fibrinolysis system [6]. Furthermore, coagulation function regulation entails multi-faceted crosstalk among anticoagulant, procoagulant, and fibrinolytic pathways. Neither a single marker nor static testing can offer a complete picture of this intricate network [7].

Antithrombin III (ATIII), the key functional molecule in the body's intrinsic anticoagulant pathway, achieves its inhibitory function by binding to proteases like thrombin and factor Xa. Lowered ATIII levels can directly bring about a relative increase in procoagulant activity, a condition that is intimately connected to the risk of recurrent IS [8]. Thromboelastography (TEG), on the other hand, offers dynamic, whole-blood-based profiling of coagulation from clot initiation, clot formation, fibrin cross-linking, and fibrinolysis, serving as a reference standard for real-time global assessment [9]. Yet, the combined potential of ATIII and TEG-derived parameters remains unexplored for constructing a predictive model of IS recurrence risk after DAPT, potentially due to the technical and logistical challenges of integrating these distinct assays.

In light of the current research status, this study develops a risk prediction framework based on the perspective of »anticoagulation-coagulation dynamic equilibrium«. Firstly, it includes ATIII as a quantitative measure of the body's endogenous anticoagulant capacity, filling the void in traditional models regarding the assessment of »anticoagulant defects«. Secondly, through the integration of TEG-based multi-parameter dynamic analysis of the coagulation and fibrinolysis processes, it realizes the accurate quantification of imbalances in »procoagulation versus anticoagulation« and »coagulation versus fibrinolysis«. This research aims to transcend the constraints of one-dimensional and static monitoring by developing dynamic risk prediction tools. Such tools will provide clinicians with a more holistic and real-time risk profile, supporting clinicians in recognizing patients at elevated recurrence risk post-DAPT and guiding personalized treatment adjustments.

## Materials and methods

### Research design

This study recruited IS patients admitted to our medical institution between May 2024 and May 2025 as the study participants. A priori sample size estimation was performed utilizing a logistic regression model via PASS 2023 software (NCSS, LLC). Based on an anticipated event rate of 18% for the primary endpoint (3-month post-DAPT recurrence), derived from prior research [10], and 20 candidate predictors (including demographics [e.g., age, sex], risk factors [e.g., hypertension, diabetes, hyperlipidemia], clinical metrics [e.g., NIHSS score], baseline laboratory parameters [e.g., platelet count, INR], ATIII activity, and thromboelastography parameters [R-time, K-time, α-angle, MA, LY30]), the requirement of 10-15 events per predictor variable indicated a need for 180-270 patients. After adjusting for an estimated 20% attrition due to factors like loss to follow-up, a final sample of 200 subjects was established. Eligibility Criteria for Inclusion: (1) Age 18 years or older; (2) Confirmed acute IS of non-cardioembolic origin (with an identified etiology other than cardiogenic embolism or small vessel occlusion); (3) DAPT initiation ≤72 hours post-onset; (4) consent and capacity for 3-month follow-up; (5) No significant coagulation impairment at baseline, defined as International Normalized Ratio (INR) ≤ 1.5 and platelet count ≥ 100x10^9^/L. Criteria for Exclusion: (1) Concurrent malignant neoplasms (<3-month survival) or hematologic disorders; (2) Major surgical procedures (e.g., craniotomy, cardiac stent implantation) or severe trauma in the preceding 3 months; (3) Significant hepatic/renal insufficiency, characterized by estimated glomerular filtration rate [eGFR] <30 mL/min/ 1.73 m^2^ or alanine aminotransferase/aspartate aminotransferase [ALT/AST] exceeding 3 times the normal upper limit; (4) Pregnancy/breastfeeding; (5) Hypersensitivity to aspirin or any P2Y_12_ receptor antagonist; (6) Pre-existing intracranial severe hemorrhage (including cerebral hemorrhage, subarachnoid hemorrhage) or active gastric ulcers; (7) Failure to comply with TEG or ATIII detection procedures due to technical issues (e.g., sample hemolysis, equipment failure).

### Data collection and detection

Baseline patient information, encompassing demographics, risk factors, and prior medications, was documented at enrollment. Meanwhile, tests for plasma ATIII, TEG examinations, and evaluations of coagulation function were administered during the 24-72 hour window after DAPT commencement.

ATIII measurement: Using the STA® Stachrom ATIII kit (Diagnostica Stago, France) on a STA-R Evolution analyzer, following plasma separation via centrifugation (3000 X g, 10 min, 4°C), the upper plasma layer was immediately assayed. Then came the sequential addition of plasma (20 μL), buffer (180 μL), and thrombin/FXa reagent (50 μL), as per the kit instruction manual, for a 5-minute incubation at 37°C. Subsequently, chromogenic substrate (50 pL) was introduced to incubate for 10 minutes at 37°C. The reaction was then stopped with 50 μL of termination reagent. Absorbance (A) readings at 405 nm were used to calculate A (sample A - blank A). Each batch included quality controls (Level 1 normal and Level 2 abnormal), requiring adherence to ±2SD acceptance criteria following Westgard guidelines.

TEG: Whole blood was analyzed utilizing the ROTEM® sigma device (Instrumentation Laboratory, USA). Key parameters were documented: reaction time (R-time, min), kinetic time (K-time, min), coagulation angle (α-angle, degrees), maximum amplitude (MA, mm), and lysis at 30 minutes (LY30, %). Each parameter reflects a specific phase of the clot formation and lysis process: R-time (clot initiation), K-time and α-angle (clot kinetics), MA (clot strength), and LY30 (fibrinolysis).

Coagulation function: The assessment was performed with an ACL TOP 700 automated coagulation analyzer. Following sample loading, plasma was automatically aspirated and analyzed for activated partial thromboplastin time (APTT), prothrombin time (PT), thrombin time (TT), fibrinogen (FIB), and D-Dimer. For quality assurance, control samples were run in each batch, with acceptable results defined as being within ±1.5 standard deviations of the established target.

### Grouping and follow-up

Standard DAPT was administered to every patient, with a treatment duration of at least 3 months (to be adjusted based on clinical status). Patient compliance was monitored through medication diaries reviewed during outpatient visits and pill counts conducted during telephone follow-ups. Follow-up was carried out via biweekly outpatient reevaluations, weekly telephone check-ups, and access to patients' hospitalization records. Endpoint events were defined as recurrent ischemic stroke (including TIA) or non-fatal myocardial infarction, encompassing newly occurred ischemic stroke (TIA included) and non-fatal myocardial infarction within the 3-month period after DAPT was started.

### Ethical approval and quality control measures

This study received formal approval from the Ethics Committee of our hospital. All patients provided written informed consent before enrollment. All testing procedures were executed by the hospital's laboratory in strict adherence to the instructions provided with the kits. Two researchers, who had received proper training and were blinded to the study group allocation and endpoint adjudication during data collection, independently collected the data.

### Statistical analysis

Statistical processing was carried out using SPSS 26.0. Normality was assessed using the Shapiro-Wilk test, descriptive statistics included means ± standard deviations (normally distributed) or medians (with 25th and 75th percentiles; non-normally distributed) for continuous measures, and counts (percentages) for categorical measures. For missing values (proportion <5%), the Multiple Imputation by Chained Equations (MICE) approach was utilized for imputation. Group comparisons utilized Student's t-test for parametric continuous data, the Mann-Whitney U test for non-parametric continuous data, and the chisquare (χ^2^) test for categorical variables. Predictor selection involved a two-step approach: univariate screening (P<0.05) logistic regression analysis (P<0.05). A final logistic regression-based model predicting IS recurrence was developed, in which recurrent events functioned as the dependent variable and key predictors as independent variables. Model performance was evaluated by the area under the receiver operating characteristic (ROC) curve (AUC) for discrimination for prediction accuracy. P-values <0.05 were deemed significant.

## Results

### Baseline patient characteristics and event occurrence

Among the 200 enrolled non-cardiogenic IS patients, the mean age stood at approximately 66.66±4.97 years, with males accounting for around 60.00% (120/200). High prevalence rates of hypertension (53.50%), hyperlipidemia (24.00%), and diabetes mellitus (41.00%) were noted. The dominant DAPT regimen was aspirin (100 mg once daily) plus clopidogrel (75 mg once daily), representing 84.00% (168/200) of the cases, with ticagrelor plus aspirin employed for the remainder (16.00%). Within 3 months after the initiation of DAPT, the incidence of recurrent events reached 21.00% (42/200).

### Univariate analysis

Patients with recurrence demonstrated markedly lower ATIII activity than the non-recurrence cohort (P<0.001), potentially linking an endogenous anticoagulant defect to a greater likelihood of recurrence. Meanwhile, TEG results showed a higher LY30 and lower MA in the recurrence group (P<0.001), pointing to a possible disturbance in the coagulation-fibrinolysis equilibrium. Additionally, coagulation tests revealed prolonged APTT and PT, along with elevated D-Dimer levels in relapsed patients (P<0.05) (Table 1).

**Table 1:** Univariate analysis of factors influencing IS recurrence.

		Non-recurrence	Recurrence (n=42)	Test statistic (t or χ^2^)	P
Age		66.42±4.15	67.57±7.27	1.340	0.182
Sex	male	94	26	0.080	0.777
	female	64	16		
BMI (kg/m^2^)		23.36±1.95	23.56±2.50	0.560	0.576
Smoking	yes	46	14	0.281	0.596
	no	112	28		
Drinking	yes	28	10	0.799	0.371
	no	130			
Complicating<br>disease	diabetes mellitus	62 (39.42)	20 (47.62)	0.963	0.327
	hypertension	82 (51.90)	25 (59.52)	0.776	0.379
	hyperlipidemia	36 (22.78)	12 (28.57)	0.609	0.435
Types of IS	large-artery atherosclerosis	112	31		
	small-vessel occlusion	31	8		
	other	15	3		
TEG	R (min)	6.34±2.39	7.00±2.64	1.549	0.123
	K (min)	2.35±0.85	2.36±0.88	0.018	0.985
	a (°)	63.45±6.48	62.52±6.07	0.844	0.400
	MA (mm)	54.44±7.23	48.03±6.64	5.189	<0.001
	LY30 (%)	2.32±0.84	3.36±1.20	6.506	<0.001
Coagulation<br>function	APTT (s)	32.52±4.85	33.77±6.79	1.357	0.176
	PT (s)	12.51±2.84	13.20±2.63	1.415	0.159
	FIB (g/L)	3.48±0.87	3.67±0.83	1.229	0.221
	D-Dimer (mg/mL)	1.19±0.40	1.73±0.80	6.104	<0.001
ATIII (%)		84.17±7.73	73.29±8.07	8.029	<0.001

### Key predictor screening

Collinearity indicated no significant multi-collinearity among the aforementioned predictive factors [variance inflation factor (VIF) <5]. Subsequent logistic regression analysis revealed that ATIII and TEG-LY30 all served as independent risk factors for IS recurrence post-DAPT, while TEG-MA independently conferred protection against recurrence (P<0.05) (Table 2).

**Table 2:** Multivariate analysis of factors influencing IS recurrence.

	B	S.E.	Wald χ^2^	P	OR	95%CI	
						Lower limit	Upper limit
TEG-LY30	-0.163	0.042	15.127	<0.001	0.85	0.783	0.922
TEG-MA	1.689	0.372	20.657	<0.001	5.413	2.613	11.214
D-Dimer	0.821	0.461	3.172	0.075	2.272	0.921	5.608
ATIII	-0.207	0.042	23.778	<0.001	0.813	0.749	0.884

### Establishment and performance assessment of the risk model

**Figure 1:**
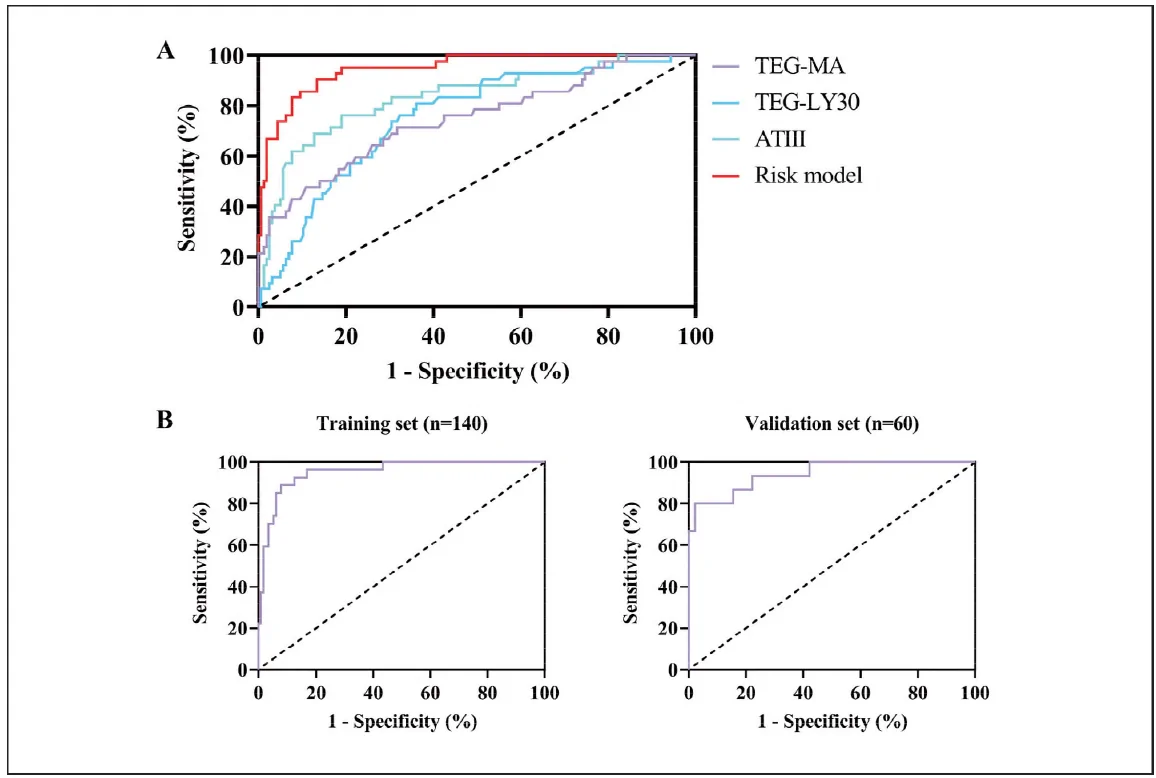
Development and validation of the IS recurrence risk model. (A) A risk model was developed based on the results of the regression analysis. (B) The performance of the model in predicting IS recurrence was verified by training set and validation set (results of ROC curve analysis).

The developed risk model, derived from regression analysis, after excluding D-Dimer, effectively predicted relapse post-IS therapy [Model = 19.376 + 1.777xATIII + (-0.222)xTEG-LY30+(-0.164)xTEG-MA]. Evaluation using ROC curves showed high predictive accuracy (sensitivity 90.48%, specificity 86.71%), with an AUC of 0.9480 (95% CI = 0.9148-0.9813), markedly outperforming predictions based on single variables (Figure 1A). This model was subsequently validated by randomly allocating all patients into a training set (n = 140) and a validation set (n = 60) at a 7:3 ratio. Consistent performance was observed, as the model achieved AUC scores of 0.9521 in the training set and 0.9437 in the validation set (Figure 1B), underscoring its potential value for clinical application (Table 3).

**Table 3:** Performance of the risk model in predicting IS recurrence.

	AUC	SE	95%CI	Sensitivity (%)	Specificity (%)	P
Model	0.9480	0.0170	0.9148~0.9813	90.48	86.71	<0.001
Training set	0.9521	0.0198	0.9132~0.9911	88.89	92.04	<0.001
Validation set	0.9437	0.0333	0.8784~1.0000	80.00	97.78	<0.001

### Subgroup analysis

**Figure 2:**
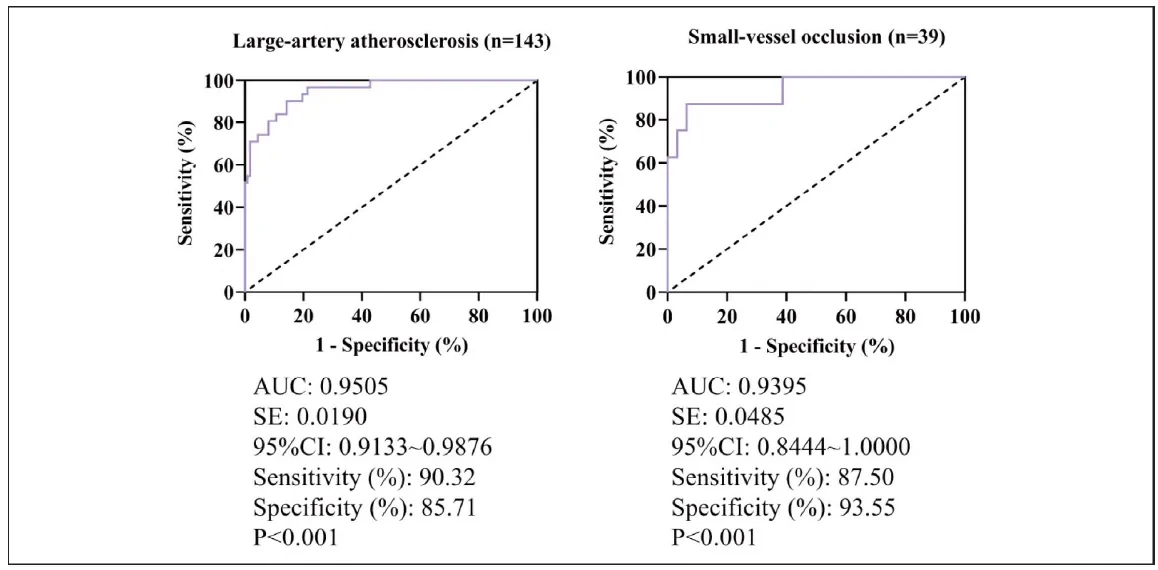
Evaluation effect of risk model in large-artery atherosclerosis subtype and small-vessel occlusion subtype.

When stratified by the types of IS, the model exhibited strong predictive capabilities across subgroups. It achieved an AUC of 0.9505 in the large-artery atherosclerosis subtype (n = 143) and an AUC of 0.9395 in the small-vessel occlusion subtype (n = 39). However, the limited sample size of the other/undetermined etiology subgroups (n = 18) precluded a reliable assessment of model performance in these categories (Figure 2). Therefore, the results for these subgroups should be interpreted with caution due to the small sample size.

## Discussion

By leveraging ATIII and TEG, this study successfully constructed a risk model for recurrence in DAPT-treated IS patients. The model, incorporating ATIII activity, TEG-LY30, and TEG-MA, showed demonstrated high discriminative ability (AUC = 0.948) in assessing IS recurrence, with performance surpassing single-parameter assessments. Internal validation (training AUC = 0.9521; validation AUC = 0.9437) further verified its generalization capacity. This model provides a scientific rationale for »personalized antithrombotic strategy« strategies, enabling a move away from empirical treatment towards personalized secondary prevention strategies for IS.

This study is the first to identify reduced ATIII activity as an independent risk factor for post-DAPT recurrence in IS patients. ATIII, a key serine protease inhibitor in the endogenous anticoagulation system, exerts its effect primarily by neutralizing thrombin and factor Xa. A reduction in its activity thus tilts the hemostatic balance toward a prothrombotic state [11][12]. Previous research has linked ATIII deficiency to atherosclerotic plaque instability, possibly by facilitating microthrombosis that exacerbates neural injury [13]. Our data, which align with reports from Luo JQ et al. [14] and extending these findings specifically to the context of recurrence risk under DAPT treatment in IS patients, show statistically reduced ATIII activity in patients with recurrence, underscoring its utility as a sensitive biomarker for gauging the compensatory state of the body's anticoagulant pathways. In addition, TEG parameters show unique predictive advantages in the model. An increased LY30 suggests overactivation of the fibrinolytic system, potentially linked to a cascading fibrinolytic response following plaque rupture [15]. A decreased MA value points to insufficient coagulation clot strength, serving as an indirect indicator of inadequate inhibition of platelet function or reduced fibrinogen concentrations [16]. In this study, the recurrence group showed a higher TEG-LY30 and a lower MA compared to the non-recurrence group, consistent with the »coagulation-fibrinolysis imbalance« theory proposed by Wang Y et al. [17]. It is worth noting that although tracking the dynamic changes in TEG parameters (such as R-time trends) could be more informative than isolated measurements, the study's design, constrained by the follow-up interval, precluded a detailed investigation into this aspect. Future studies incorporating repeated TEG assessments at predefined intervals (e.g., weekly or biweekly) are warranted to explore the prognostic value of temporal parameter evolution.

The combined utilization of ATI 11 and TEG parameters in the model reflects the coordinated regulatory mechanism underlying the »anticoagulation-coagulation-fibrinolysis« network. The interplay between reduced ATI 11 activity (weakening endogenous anticoagulant barrier) and increased TEG-LY30 (signaling hyperfibrinolysis) exemplifies the breakdown of coagulation homeostasis. The concomitant elevation of D-Dimer, a fibrinolytic product, validates this fibrinolytic activation [18]. This novel multidimensional paradigm addresses the complexity of poststroke hypercoagulability more effectively than traditional single-index methods (e.g., NIHSS). Notably, the inverse D-Dimer/TEG-MA correlation hints at fibrinolysis indirectly impairing clot strength via fibrinogen degradation, partially validating the »antifibrinolytic therapy window« hypothesis. Moreover, the model's integration of biomarkers and dynamic coagulation testing provides a real-time perspective missing from previous approaches, enhancing its superior clinical decision-support capabilities.

In future clinical management of IS, we propose proactive screening for ATIII activity and TEG parameters before initiating DAPT Individuals identified as high-risk (ATIII activity <84% plus TEG LY30 >2.3%) should be considered for an extended DAPT course of six months, supplemented with bridging low-molecular-weight heparin (LMWH). During DAPT administration, weekly TEG monitoring is recommended. A rising trend in MA coupled with a declining LY30 portends an elevated risk of recurrent thrombosis due to impaired fibrinolysis. In such cases, switching to ticagrelor or adding cilostazol may be considered. Meanwhile, integrating the ATIII-TEG model into the electronic medical record (EMR) system is a viable consideration, subject to addressing potential challenges related to healthcare infrastructure, data privacy regulations, and implementation costs. By leveraging artificial intelligence algorithms, this integration could enable real-time risk alerts, thereby enhancing the risk identification capabilities of primary healthcare institutions. However, prior to its inclusion in clinical guidelines, the model must undergo external validation in multi-center studies that represent diverse racial groups. A cost-benefit analysis is also essential to justify its implementation. Furthermore, although the sample size of this study was rigorously estimated (n=200), the small number of participants in specific subgroups (e.g., other definite/unknown etiology, n = 20, as shown in Figure 2) could limit the external validity of the results. The single-center design also raises the possibility that patient baseline features may differ from those in real-world settings, highlighting the need for multi-center validation. Moreover, the timing of TEG and ATIII assessments was restricted to the 24-72 hour window post-DAPT initiation, leaving coagulation state changes in the early treatment period (such as within 7 days) unrecorded and potentially resulting in the omission of significant hemodynamic shifts. Subsequent investigations are warranted to thoroughly explore these constraints.

## Conclusion

A novel model is established and validated to assess the risk of IS recurrence post-DAPT. This model dynamically profiles coagulation-fibrinolysis function by integrating plasma ATIII activity with TEG parameters, achieving an AUC increase of 0.15-0.20 compared to NIHSS-based predictions. Its application could facilitate more precise stratification and management in secondary prevention of IS. Future efforts are needed to verify its universality in multi-center, large-scale cohorts and investigate how this model interacts synergistically with emerging antithrombotics like tirofiban. The ultimate goal is to guide its transition from experimental validation to clinical application.

## Dodatak

### Availability of data and materials

The data that support the findings of this study are available from the corresponding author upon reasonable request.

### Conflict of interest statement

All the authors declare that they have no conflict of interest in this work.
